# Influence of TikTok on Body Satisfaction Among Generation Z in Indonesia: Mixed Methods Approach

**DOI:** 10.2196/58371

**Published:** 2024-09-06

**Authors:** Hanifa Ariana, Ikmal Almuhtadi, Nikita Jacey Natania, Putu Wuri Handayani, Stéphane Bressan, Pramitha Dwi Larasati

**Affiliations:** 1 Faculty of Computer Science Universitas Indonesia Depok Indonesia; 2 School of Computing National University of Singapore Singapore Singapore

**Keywords:** body satisfaction, social media, TikTok, Indonesia, cyber-bullying, cyberbullying, cyberbully, cyber-harassment, bullying, harassment, body shaming, objectify, objectifying, social media, social media use, social media usage, socials, social network, social networks, social networking, Tik Tok, GenZ, Gen-Z, youth, adolescent, adolescents, teen, teens, teenager, teenagers, young-adult, young-adults

## Abstract

**Background:**

As social media platforms gain popularity, their usage is increasingly associated with cyberbullying and body shaming, causing devastating effects.

**Objective:**

This study aims to investigate the impact of social media on Generation Z users’ body image satisfaction. More specifically, it examines the impact of TikTok on body image satisfaction among TikTok users aged between 17 years and 26 years in Indonesia.

**Methods:**

The methodology used mixed-method approaches. Quantitative data were obtained from 507 responses to a questionnaire and analyzed using covariance-based structural equation modeling. Qualitative data were obtained from the interviews of 32 respondents and analyzed through content analysis.

**Results:**

This study reveals that upward appearance comparison is influenced by video-based activity and appearance motivation. Conversely, thin-ideal internalization is influenced by appearance motivation and social media literacy. Upward appearance comparisons and thin-ideal internalization comparisons detrimentally impact users’ body image satisfaction.

**Conclusions:**

The results of this study are expected to provide valuable insights for social media providers, regulators, and educators in their endeavors to establish a positive and healthy social media environment for users.

## Introduction

### Background

In 2022, the estimated number of social media users was 4.80 billion, with an expected increase to 7 billion by the decade’s end [1]. Concomitantly, the use of social media appears to be increasingly linked to cyberbullying, especially in the context of body shaming—the act of criticizing and stigmatizing someone’s physical appearance [2]. Noteworthy cases involving public figures such as Kylie Jenner and Adele [3,4] underscore the fact that nobody is exempt from such incidents.

Individual perceptions of one’s body can range from positive to negative [5], with positive perceptions indicating body satisfaction (BS) and negative perceptions indicating body dissatisfaction. Body image, including BS or dissatisfaction [6], plays a pivotal role in various aspects of psychological development, interpersonal relationships, and overall quality of life [6,7]. A survey of 5623 adolescents and adults in England by the UK Mental Health Foundation [8] shows that 1 in 5 individuals felt ashamed of their body image, resulting in feelings of anxiety, depression, and even suicidal thoughts. Disturbances in body image perception, such as body dissatisfaction, are pertinent factors in clinical issues, including obesity and eating disorders [6]. Generally, body image perception is an indicator of quality of life [7]. Consequently, understanding the relationship between social media use and body image is essential for addressing these significant issues.

While previous studies have explored the influence of appearance, family, peer, and media pressures on body dissatisfaction among teenage girls (eg, work by Roberts et al [9]), there has been a notable absence of specific attention to the impact of social media activities or aspects on body dissatisfaction. Conversely, Rodgers et al [10] found a connection between social media use, upward appearance comparison (UAC), and body dissatisfaction, with the latter being mediated by the internalization of an idealized appearance. Upward social comparison typically involves evaluating oneself with the aim of self-improvement by assessing the perceived advantages of the objects being compared and learning ways to enhance one’s own attributes. Meanwhile, downward comparison is the process of comparing oneself with someone judged to be worse than oneself. Upward social comparison is often deemed unfavorable since the assessed object holds a higher “value” than oneself, while downward comparison is considered favorable as the object holds a lower “value” than oneself [11]. Therefore, this research will primarily focus on the dynamics of upward comparisons.

Furthermore, prior research has predominantly focused on platforms such as Snapchat, Facebook, and Instagram [9,12,13]. Studies related to TikTok delve into areas such as body dissatisfaction [14,15], BS in dancer challenges [16], body neutrality [17], and systematic literature reviews on body image [18]. TikTok has a substantial user base primarily consisting of Generation Z (Gen Z) individuals, aged between 11 years and 26 years, with approximately 37 million users worldwide. Indonesia stands as the second largest TikTok user in the world to date [19,20]. Finally, TikTok is one of the social media that focuses on user-generated videos that display the appearance of the human body, such as content in the category’s selfie videos, self-portrait videos, dance, fashion, beauty and skin care, fitness or sports, and entertainment [21]. TikTok also strives to create an environment to support user’s body positivity [21].

### Conceptual Model

Jarman et al [12] applied the tripartite influence model (TIM) to investigate the relationship between social media intensity, frequency of use, BS, and well-being mediated by internalization and comparison in Australia. However, their study did not explicitly consider content related to appearance. This study uses the TIM, a sociocultural theory that explores the influence of family, friends, and media on body image dissatisfaction and eating disorders. To narrow the focus to social media use, we chose to emphasize 1 sociocultural aspect—social media. TIM suggests that the stimulus aspect has a notable impact on physical appearance [14]; yet, it does not thoroughly address how social media specifically affects body image. To address this gap, our focus lies on social media use involving videos prominently featuring physical appearance. Moreover, we use stimulus-organism-response (SOR) theory to categorize factors into stimulus, organism, and response. The SOR framework states that environmental conditions can present signs that can trigger (stimulus) the inner state of an individual (organism) so that the individual will produce a response (response) in either positive or negative form [22]. The development of SOR theory can help in understanding the reasons behind a person’s behavior with the assumption that most human behavior reflects the stimuli we feel, so this theory is very relevant when it is related to problems related to human behavior under certain conditions. The combination of SOR and TIM offers a more comprehensive context for understanding the influence of social media use on human BS, using the explanatory power of SOR theory to delve into the reasons behind human behavior (response) in specific situations.

By focusing specifically on TikTok, we aim to identify the factors within this social media platform that influence BS among Gen Z individuals. Through this investigation, our goal is to shed light on the factors that contribute to body shaming incidents, increase user awareness of the effects of social media on body image perception, and empower individuals to take control of their usage. These insights can also serve as valuable guidance for social media developers, aiding them in creating platforms that foster a positive digital environment. Moreover, stakeholders can use these findings to inform and shape regulatory efforts, contributing to the establishment of a safer digital space.

According to TIM, pressure related to appearance is a consequence of sociocultural factors [9]. Therefore, the use of social media, especially on platforms such as TikTok, offers a means to assess these sociocultural influences. TikTok, being a platform centered around appearance, typically entails activities oriented toward videos (referred to as video-based social media use [VBA]), such as viewing, uploading, and interacting with images of oneself and others [23]. Examining specific activities to measure social media use can facilitate a deeper understanding of how social media affects body image [12].

**Figure 1:**
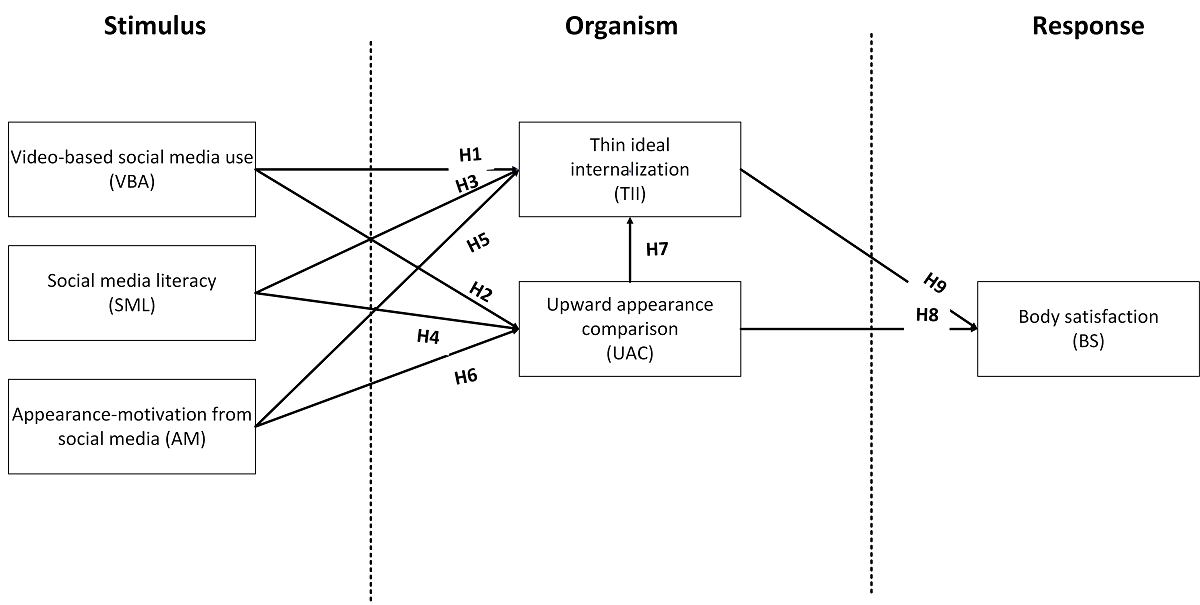
Proposed conceptual model. AM: appearance-motivation from social media; BS: body satisfaction; H: hypothesis; SML: social media literacy; TII: thin-ideal internalization; UAC: upward appearance comparison; VBA: video-based social media use.

Mink and Szymanski [14] suggested that variables of social media literacy (SML) could moderate the impact of TikTok usage on body dissatisfaction. Similarly, Tort-Nasarre et al [24] highlighted the importance of this variable in research concerning the impact of social media use on BS, noting that a high level of SML can assist individuals in developing positive body perceptions in conjunction with social media use. Furthermore, Roberts et al [9] indicated that internalization encompasses various aspects, such as thin-ideal internalization (TII), muscular internalization, and social media internalization. For our purposes, we narrow our focus to TII and social media internalization, as these variables are more inclusive of both genders than the more gender-specific muscular internalization. Thus, we propose Figure 1 as our conceptual model.

### Research Hypothesis

The activities examined in this study pertain to video-related actions because TikTok is a social networking site centered around video-based appearances [14]. According to Mink and Szymanski [14], these activities allow users to engage with the physical representation of other people’s lives, including their appearance, through features such as share and comments. TII is an individual’s cognitive level to believe and inspire that the ideal social definition of attractiveness, namely a thin appearance, is an actual attractiveness [25]. Jarman et al [12] stated that the use of social media, including the activities carried out on it, is one of the reasons for the internalization of an ideal thin appearance. Jarman et al [12] found that the more frequent use of social media led to a higher likelihood of internalizing an ideal thin appearance. In addition, Duan et al [26] found that people who are involved in activities on social media are more likely to internalize their ideal appearance; in this case, they internalize their appearance to make themselves look thin. Such internalization is made possible because videos posted on social media, such as selfies, can be seamlessly modified and edited using filters, artificial intelligence, and image processing tools and uploaded selectively by people to meet social body standards [27,28]. Considering various activities on social media, such as viewing posts or comments portraying ideal physical appearances on platforms such as TikTok, users are likely to internalize the notion of an ideal thin appearance. Therefore, we assume the following hypothesis:

H1: VBA influences TII.

Users engaging in VBA activities have several goals, such as admiring individuals whose physical appearance aligns with societal standards, maintaining relationships, and receiving visual feedback [29]. However, many studies have found that user participation in activities on social media, such as scrolling through posts adhering to ideal standards [30], uploading content with positive comments [31], and content receiving numerous likes [32], can negatively impact individual perceptions of body image. As outlined by Mink and Szymanski [14], activities within VBA platforms allow users to be involved with the physical representation of others’ lives, including their appearance, through features such as likes, shares, and comments. UAC is an individual behavior where one assesses their appearance against individuals deemed to possess a superior appearance, such as celebrities or social media influencers [33]. According to Wang et al [34], social media users who engage in appearance comparisons often do so with individuals considered superior, especially those portraying an idealized life on social media. This comparison prompts individuals to seek improvement, often leading to the editing of selfies to compensate for perceived weaknesses [27]. Women, in particular, may seek quantitative proof of popularity through metrics such as likes, comments, and followers [28]. After reviewing several previous studies, we posit that UACs, including those with celebrities or influencers, are also influenced by VBA usage activities. When TikTok users encounter videos showcasing an ideal physical appearance uploaded by influencers or celebrities, accompanied by positive reactions from other users, they are likely to initiate comparisons of their own physical appearance with these influencers or celebrities. Therefore, we propose the following hypothesis:

H2: VBA influences UAC.

SML is the application of critical analysis regarding the motivations behind postings on social media and the nature of images that are constructed, generally unrealistically, when viewing images that focus on appearance on social media [35]. According to Tamplin et al [36], a high level of media literacy is characterized by the ability to think critically about media. Tamplin et al [36] explained that the ability to critique idealized performance images, considering their realism and commercial intent, helps offset the impact of viewing these images on individuals. The level of media literacy is also marked by the ability to judge whether or not an image in the media is intended to reduce the credibility and persuasive influence of the media [37]. Individuals with high levels of media literacy tend to avoid comparing themselves with ideal thin appearances in media that are considered unrealistic [37]. McLean et al [37] also found that media literacy produces a protective effect, in which research respondents who have a low level of critical thinking are easily negatively affected when viewing images that display someone who has an ideally thin appearance, and research respondents who have high critical thinking are not easily negatively affected. Therefore, we propose the following hypothesis:

H3: SML influences TII.

McLean et al [37] explained that individuals with high media literacy tend to prefer to compare themselves with ideal thin appearances in the media, which are considered unrealistic. A high level of media literacy, such as thinking that postings of someone whose physical appearance looks ideal in the media are unrealistic or manipulated, can protect users from upward comparisons, such as those with celebrities or influencers [37]. McLean et al [37] also stated that high critical thinking is a protective factor from a high upward comparison. Mink and Szymanski [14] also found that SML exacerbated the direct relationship between TikTok use and UACs. When users have low SML skills, they may not be able to determine the authenticity of the TikTok videos they see. They may need to realize that the videos have been edited or changed [14]. Therefore, we assume the following hypothesis:

H4: SML influences UAC.

Appearance motivation (AM) from social media is a feeling that arises when individuals want to look attractive and try to improve themselves because they are motivated by messages conveyed by the media [10]. The media plays an important role in spreading the message that an ideal appearance is a good thing, such as popularity, and success can be achieved if someone has an attractive appearance [38]. Attractive appearance in the media has standards, such as that women must be young, thin, and sexy [38]. When the message reaches the audience, they will become motivated and try to change their appearance to make it look more ideal [38]. In addition, Kvardova et al [39] explained that the effects of media use tend to affect the internalization of an ideal thin appearance. In line with Wilson et al [40], internalizing an ideal thin appearance can be interpreted as an indicator of a motivational approach to thin bodies. According to Graff [41], social media displays content that is somewhat unrealistic and causes users to compare their appearance, thus motivating them to engage in diet and exercise. Therefore, we propose the following hypothesis:

H5: AM from social media influences TII.

Motivation for appearance from social media is a feeling that arises when individuals want to look attractive and try to improve themselves because they are motivated by messages conveyed by the media [10]. The motivation to improve by making comparisons based on the desire to improve aspects of oneself has been associated with increased exercise activity and BS [15]. When individuals are motivated to improve their physical appearance, they tend to make upward social comparisons [42], such as following attractiveness standards by changing their physical appearance to become ideally thin [43]. In addition, when individuals are urged to improve themselves, they will compare themselves with others who are better off, such as idealized models in the media [10]. Then, we suggest the following hypothesis:

H6: AM from social media influences UAC.

UAC indicates that individuals evaluate their appearance against other individuals who are considered to have a superior appearance than themselves, such as celebrities or social media influencers [44,45]. TII or internalization of an ideal thin appearance is an individual’s cognitive level to believe and inspire that the ideal social definition of attractiveness, namely a thin appearance, is an actual attractiveness [25]. Rodgers et al [10] found that social media use may be associated with body dissatisfaction through comparisons of upward appearance and internalization of thin appearance. According to Seekis et al [45], a comparison of upward appearances with celebrities or health influencers leads to the drive to be thin and dissatisfied, respectively. In addition, according to Hsu et al [46], internalization measures of ideal thin appearance include statements that assess the degree of comparison with an ideal thin body, such as “I wish I looked like a swimsuit model” and “I often read magazines such as Cosmopolitan, Vogue, and Glamor and comparing my looks to models.” Therefore, we assume the following hypothesis:

H7: UAC influences TII.

Social media impacts the development of body dissatisfaction through 2 channels: the comparison of appearances on social media and the internalization of ideal appearance [12]. According to Wang et al [34], social media users who compare themselves may engage in upward comparisons, considering the appearance and ideal life posted on social media, which is likely to have a detrimental effect on BS and well-being. According to the TIM, exposure to the media can increase the likelihood that individuals will adopt an ideal appearance as their standard and contribute to a greater tendency to compare their physical appearance with someone’s unrealistic uploads in the media [12]. Individuals who compare themselves with social media’s ideal physical appearance standards will likely feel that they do not conform to them, reducing their BS [12]. Mink and Szymanski [14] showed that the more often individuals use TikTok, the more they engage in appearance comparisons and the more body dissatisfaction they experience. Today, individuals have access to people worldwide, meaning that comparisons can occur at an unprecedented level and scale [47]. Thus, we assume the following hypothesis:

H8: UAC influences BS.

TII or internalization of an ideal thin appearance is an individual’s cognitive level to believe and inspire that the ideal social definition of attractiveness, namely a thin appearance, is an actual attractiveness [36]. Individuals often post carefully edited and curated content about their physical appearance, which presents unrealistic content to make them appear attractive [25]. Rodgers et al [10] stated that individuals involved in internalizing an ideal appearance have a lower level of performance satisfaction. Individuals use media content as a source of information about how to improve their physical appearance and compare their physical appearance with media models to set standards that must be met [10]. Jarman et al [12] also found that internalizing an ideal thin appearance is inversely related to BS. Exposure to images on social media internalizing an ideal thin appearance causes dissatisfaction with the body and face of the individual [32]. Thus, we propose the following hypothesis:

H9: TII influences BS.

## Methods

### Study Design

This study used a mixed methods approach using questionnaires (quantitative approach) and interviews (qualitative approach). A readability test was carried out on the questionnaire to verify the appropriateness of the questions within the research context. This assessment encompassed an examination of the diction and grammar used in the questionnaire to ensure clarity. The primary aim was to ascertain that all questions and instruments in the questionnaire could be easily and clearly understood by the respondents. The readability test was carried out for 6 days with a total of 13 respondents. This evaluation took place through both digital and offline channels. We conducted a pilot study of 33 respondents where the Cronbach α (CA) value for each construct was more than 0.7 [48].

The questionnaire link was distributed through various social media, such as Instagram, Twitter, Line, and WhatsApp, from February 28 to March 29, 2023, with a total duration of 30 days. Respondents involved in this study are TikTok users who are Gen Z (aged between 17 years and 26 years). Most of the respondents are in Greater Jakarta and are bachelor students. Most users’ frequent activities on TikTok are liking videos and they often view content related to physical appearance as well as seeing body shaming comments on TikTok. This study obtained 507 respondents, and Table 1 summarizes the demographics of the respondents. After obtaining quantitative data, we collected qualitative data by conducting interviews with 32 TikTok users in the age range of 17-26 years.

**Table 1:** Demographics of respondents who filled out the questionnaire.

Demographics			Respondents, n (%)
**Gender**			
	Men	134 (26)	
	Women	373 (74)	
**Age (years)**			
	17-19	76 (14.99)	
	20-22	339 (66.86)	
	22-26	92 (18.15)	
**Domicile**			
	Greater Jakarta	312 (61.54)	
	Java Island outside Greater Jakarta	125 (24.65)	
	Outside Java Island	70 (13.81)	
**Education**			
	High school students	93 (18.34)	
	Diploma students	45 (8.88)	
	Bachelor students	355 (70.02)	
	Master students	14 (2.76)	
**Reasons to use TikTok**			
	Entertainment	421 (47.09)	
	Increase creativity	129 (14.43)	
	Know the latest information	301 (33.67)	
	Add friendship	29 (3.24)	
	Others	14 (1.57)	
**Most frequent activities on TikTok**			
	Upload a video	128 (18.47)	
	Comment on a video	132 (19.05)	
	Like a video	433 (62.48)	
**Frequency of viewing content related to physical appearance on TikTok**			
	Never	15 (2.96)	
	Rarely, occasionally when using TikTok	244 (48.13)	
	Often, every time TikTok is used	248 (48.92)	
**Frequency of seeing body shaming comments on TikTok**			
	Never	51 (10.06)	
	Rarely, occasionally when using TikTok	220 (43.39)	
	Often, every time TikTok is used	236 (46.55)	

Quantitative data for this study were processed using the covariance-based structural equation method (CB-SEM) using the AMOS 24 (IBM) app, SPSS Statistics (version 27; IBM Corp), Google Sheets, and Excel (Microsoft Corp). CB-SEM aimed to confirm the relationship between variables defined theoretically in a model, attempting to minimize the differences between the covariance matrices implied in the model and the covariance matrices obtained from research samples [48]. The CB-SEM procedure included 3 stages: model specification, measurement model evaluation, and structural model evaluation [48]. In addition, content analysis was used to process qualitative data.

To analyze in more detail the results of hypothesis testing, interviews were conducted with respondents aged between 17 years and 26 years who had at least actively used the TikTok application. A total of 32 respondents were interviewed from April 19, 2023, to May 13, 2023, digital and offline. All interview results obtained were then processed and analyzed using one of the qualitative data processing methods, namely content analysis. This stage produces an interpretation of all patterns resulting from the interview process so that it can support the explanation of the hypothesis results in this research.

### Research Instruments

The questionnaire was composed of 4 parts. The first part contained instructions for filling out the questionnaire, the respondent’s consent, and validation that the respondent is a TikTok application user and is aged 17-26 years. The second part contained questions on the demographics of the respondents. The third section contained general information about the TikTok application, physical appearance content, and body shaming. The fourth section contained 32 measurement items. Multimedia Appendix 1 describes the questionnaire used in this study, and Multimedia Appendix 2 explains the interview questions for each hypothesis.

### Ethical Considerations

This study has obtained approval from the research unit of the Faculty of Computer Science, University of Indonesia (letter S-1/UN2.F11.D1.5/PPM.00.00/2024). In accordance with the Decree of the Board of Trustees of the University of Indonesia, based on the research ethics policies applicable to members of the faculty of the University of Indonesia, each research unit or department implements research supervision guidelines, and oversees and adheres to the responsibilities prescribed therein. In line with university policy, the Research and Community Service Department, Faculty of Computer Science, University of Indonesia, adhere to the guidelines and procedures established by the faculty and provided ethics approval for this study. All respondent data were anonymized and exclusively used for the purposes outlined in this research. All questionnaire respondents provided written informed consent, and all the interview respondents provided verbal informed consent to participate in this study.

## Results

All variables in Table 2 surpass the 0.50 threshold of average variance extracted values, indicating the successful representation of the variables by their respective indicators. Additionally, the reliability of the measurements was assessed using CA and construct reliability. All CA and construct reliability values met the recommended minimum threshold of 0.7, signifying strong reliability [48]. Goodness-of-fit index values are outlined in Table 3, while Table 4 provides an overview of the *R*^2^ values.

**Table 2:** CR^a^, CA^b^, and AVE^c^ values.

Variable	CR	CA	AVE
Video-based social media use	0.858	0.896	0.559
Social media literacy	0.788	0.826	0.712
Appearance motivation	0.917	0.888	0.948
Thin-ideal internalization	0.910	0.936	0.857
Upward appearance comparison	0.881	0.913	0.750
Body satisfaction	0.922	0.933	0.834

^a^CR: construct reliability.

^b^CA: Cronbach α.

^c^AVE: average variance extracted.

**Table 3:** Goodness-of-fit index values.

GoF^a^ index	Cutoff value	Value	Description
CMIN/df^b^	<2	1.213	Good fit
RMSEA^c^	0.08	0.021	Good fit
NFI^d^	0.9	0.967	Good fit
CFI^e^	0.9	0.994	Good fit
GFI^f^	0.9	0.95	Good fit
TLI^g^	0.9	0.992	Good fit
RMR^h^	0.05	0.05	Good fit

^a^GoF: goodness-of-fit.

^b^CMIN/df: minimum discrepancy function by degrees of freedom divided.

^c^RMSEA: root-mean-square error of approximation.

^d^NFI: normed fit index.

^e^CFI: comparative fit index.

^f^GFI: goodness-of-fit index.

^g^TLI: Tucker-Lewis index.

^h^RMR: root-mean-square residual.

**Table 4:** *R*^2^ values.

Dependent variable	*R* ^2^
Upward appearance comparison	0.451
Thin-ideal internalization	0.452
Body satisfaction	0.099

Next, the hypothesis testing carried out is a 2-tailed test. Hypothesis testing can be done by looking at the significance value or *P* value. Since the test is carried out in 2 directions, this test uses a significance of 5%, which means that a hypothesis with a *P* value of >.005 is rejected and vice versa is accepted [48]. Based on Table 5, 2 of the 9 hypotheses were rejected because they had a *P* value of >.005.

**Table 5:** Hypothesis testing results.

Hypothesis	Estimate	*P* value	Description
H1: VBA^a^ influences TII^b^	–0.082	.19	Rejected
H2: VBA influences UAC^c^	0.469	.002	Accepted
H3: SML^d^ influences TII	–0.14	.001	Accepted
H4: SML influences UAC	–0.083	.06	Rejected
H5: AM^e^ influences TII	0.261	.002	Accepted
H6: AM influences UAC	0.28	.001	Accepted
H7: UAC influences TII	0.534	.003	Accepted
H8: UAC influences BS^f^	–0.204	.003	Accepted
H9: TII influences BS	–0.168	.006	Accepted

^a^VBA: video-based social media use.

^b^TII: thin-ideal internalization.

^c^UAC: upward appearance comparison.

^d^SML: social media literacy.

^e^AM: appearance motivation.

^f^BS: body satisfaction.

This study concludes that there is no significant relationship between the activity of using VBA and the internalization of an ideal thin appearance. TikTok shares this observation by expressing its commitment to supporting existing content to be more inclusive and a “body-positive environment” [21]. Some of the steps taken by TikTok are increasing restrictions on ads that promote negative body image, reducing advertisements for diet products that exaggerate their promotion, working with creators to promote body positivity, and working with various organizations to help people who experience body image problems [21]. Respondents also felt that an ideal body does not mean thin or slender physical appearance: “In my opinion, a body that is still classified as ideal is if the body is slightly heavier than BMI. If a thin body is not ideal in my opinion” (interviewee 27) and “Body ideal is a body that is not thin and not fat” (interviewee 2). Then, respondents who feel their physical appearance is thin enough or feel their physical appearance is good enough have no desire to be thinner anymore: “Not really wanting to change their appearance to be thinner, but more motivated to change their lifestyle to be healthier” (interviewee 4). This is also encouraged by the growth of TikTok content, which promotes self-love or body positivity toward physical appearance and can improve the mental health and well-being of its users [49]. This movement changes the perspective of its users in viewing their physical appearance positively [49]. They realize that they are not alone when they feel insecure and know that many other users on TikTok are spreading this positive movement [49].

In addition, the relationship between the activity of using VBA influences the UACs. This is in line with Mink and Szymanski [14], who stated that the longer an individual uses TikTok, the more involved they are in comparing appearance and the more they will watch their body. In addition, Jarman et al [12] stated that when individuals are exposed to content on social media, the ideal body for them is the body they see. The impact is that they will compare their appearance with this ideal body perception and tend to produce negative self-evaluations [12]. Furthermore, the results of this hypothesis were strengthened by the results of qualitative interviews, which explained that respondents felt that the longer they opened TikTok, the more they would be increasingly exposed to physical appearance content that displays ideal body standards according to users. At first, they just admired, but over time, the respondents wanted to look as good as the people in the content they saw on TikTok: “If you look at a tall physical appearance, especially men, it will affect self-assessment and comparison with that person” (interviewee 20). In addition, this is exacerbated by the TikTok algorithm, which recommends videos according to the number of viewing likes: “Because I often see physical appearance content, especially for women who like to upload dance content on TikTok, I wonder why people that person is prettier than me. That person has clean skin, and I am also influenced to use skin care so that I look as good as the person I see on TikTok” (interviewee 32). This is also directly supported by TikTok, which explains that their recommendation system works after looking at user preferences through various interactions, such as comments or following certain accounts [49].

This study found that SML influences the internalization of an ideal thin appearance, which aligns with McLean et al [37]. In addition, Tamplin et al [36] explained that the ability to critique idealized performance images, considering their realism and commercial intent, helps offset the impact of viewing these images on individuals. Furthermore, the results of this hypothesis are strengthened by the results of qualitative interviews, which explain that respondents tend to be more aware of content that promotes a thin body as the ideal body, especially in less realistic content, for example, the TikTok trend to show a thin body: “If you have to the point that it is unrealistic, it is very toxic and destructive. If it is more realistic, in my opinion it is quite good, such as the promotion of a healthy lifestyle” (interviewee 19) and “This trend is not good because it sets new standards that lead to body shaming if you don't meet these standards” (interviewee 27). In addition, respondents believe more in content that promotes a slim body is an ideal body if the content creator has credibility, for example, a nutritionist and bodybuilder: “If the content is made by a health professional and can prove it, I agree with the content” (interviewee 26). This is in line with Mink and Szymanski [14], who explained that it is easy for individuals to determine the authenticity of the content they see when they have high SML.

Moreover, this study observed that there is no significant impact of SML on UACs. This is in line with Mink and Szymanski [14], where individuals who have strong SML skills can inadvertently make appearance expectations and appearance evaluations more prominent in women’s minds. Hence, women must change their physical appearance by using filters or editing to look as good as people they find attractive. The results of this hypothesis are strengthened by the results of qualitative interviews, which explain that respondents still compare their physical appearance with people they consider better looking in TikTok even though their level of SML is already high, for example, by knowing the use of filters on the videos they watch: “Ever compared because they saw other people use filters too and are curious about how they look on their own faces” (interviewee 7) and “I’ve compared if it matches my preferences. There was also a thought that I wanted to be like him because it’s more in line with preferences and just looks better” (interviewee 29).

Furthermore, this study revealed that AM from social media influences the internalization of an ideal thin appearance, and this result is in line with Trekels and Eggermont [38], where attractive appearance in the media has standards, such as that women must be young, thin, and sexy. When the message reaches individuals, they will become motivated and change their appearance to make it look better [38]. Furthermore, the results of this hypothesis are strengthened by the results of qualitative interviews, which explain that respondents are motivated after watching content that promotes better physical appearance, such as exercise and a healthy lifestyle: ”Feel motivated to look more attractive when viewing makeup, skin care, or sports content” (interviewee 5) and “Ever motivated after seeing only gym content but wasn't interested in looking thin like a model” (interviewee 1). After being motivated, the interviewees felt moved to change their physical appearance by doing the same thing to have an equally good physical appearance. In line with Trekels and Eggermont [38], the media is important in spreading the message that an ideal appearance is a good thing. For example, popularity and success can be achieved if someone has an attractive appearance.

This study affirmed that social media AM influences the UAC, which is in line with Rodgers et al [10,42], where when individuals have the drive to improve themselves, they will compare themselves with other people who are better looking for them, such as idealized models in the media. In addition, when individuals are motivated to improve their physical appearance, they tend to make upward social comparisons [42,43]. Furthermore, the results of these hypotheses were strengthened by the results of qualitative interviews, which explained that 24 out of 32 (75%) respondents felt that if they watched a video that presented an ideal physical appearance in their opinion, the respondents wanted to know whether their physical appearance was as good as the ideal standard derived from the video they watched: “Once motivated to eat healthy food made by people on TikTok, where they have more fit bodies, so they are influenced to make that food” (interviewee 5). In line with Grabe et al [43], when individuals feel motivated to improve their physical appearance, they follow attractiveness standards by changing their physical appearance.

In addition, respondents also felt that content displaying an ideal physical appearance on TikTok received validation, such as praise comments. Hence, respondents felt motivated to change their physical appearance to be more ideal: “Very motivated to look cool and handsome because people also commented that interesting in the content” (interviewee 20) and “Motivated to look more ideal because if you upload a video later the audience will definitely like it and there will be lots of laudatory comments” (interviewee 3). This aligns with Trekels and Eggermont [38], in which media messages that focus on appearance can be strengthened through validation from others, such as comments. Rodgers et al [10] also explained that comments from users on social media on content that focus on physical appearance explicitly describe appearance comparisons and can encourage other users to engage in physical appearance comparisons.

The comparison of upward appearance is found to influence the internalization of an ideal thin appearance. This is in line with the research of Seekis et al [45], where when individuals have the desire to look as good as celebrities and influencers, there is an urge to change their physical appearance to become thinner. Furthermore, the results of the hypothesis are strengthened by the results of qualitative interviews, which explain that respondents try to look as good as people they consider ideal on the TikTok app: “Ever compared with thinner people. Feeling a thin body is interesting and has quite an impact on my thoughts towards an ideal body because it indicates the person has a healthy lifestyle” (interviewee 28). They usually assess to measure how far their score is from the ideal value so that when they feel they have not met these ideal standards, they tend to internalize new ideal standards: “Ever compared and tried to measure whether my body already looks the same as his, because almost every I watch TikTok every day and see interesting people, so it has quite an influence on the perception of an ideal body and beauty standard” (interviewee 3) and “Because I compare things, I can know what the ideal point is like. If I’m not ideal, I will do certain activities so that I can be as ideal as that” (interviewee 26). This is in line with Hockey et al [50] and Shen et al [51], who stated that the effect of using social media is associated with a high level of comparison and ultimately leads to internalization of the ideal thin appearance of its users.

Next, individuals who compare themselves with ideal physical appearance standards on social media will most likely feel that they do not conform to these standards, thereby lowering their level of BS. This is in line with Weinstock [47], where individuals not only compare themselves with others, but they also compare themselves with other people’s optimized versions thanks to video editing apps, which can worsen mental health and lower the individual’s level of satisfaction. Conversely, the respondents have no desire to have the same physical appearance as the videos they watch on the TikTok app because their BS tends to be high: ”Does not affect self-satisfaction because they are already satisfied with their current physical appearance“ (interviewee 25) and “Comparing physical appearance to the one in the video doesn’t really affect self-satisfaction because you feel enough with what you are now” (interviewee 18). They already feel sufficient and confident with their current physical appearance: “I don’t compare with other people; I don’t even care about the scales either. Now, I just look in the mirror and observe whether I have approached the ideal body or not. I feel quite satisfied when, for example, I wear clothes and look good when worn” (interviewee 5). This is also in line with Rodgers et al [10], who stated that high self-confidence or self-esteem tends to result in lower levels of dissatisfaction, especially among women.

This study found that internalizing an ideal thin appearance is inversely related to BS. When individuals are not affected by internalizing the thin appearance of the content they see on social media, they tend to be satisfied with their current physical appearance [12]. Furthermore, the results of this hypothesis are strengthened by the results of qualitative interviews, which explain that 17 out of 32 (53%) respondents internalize the ideal thin body standard with what they see on the TikTok app, so most of their BS levels are also affected because they feel they have not met the ideal body standard. This is in line with Tiggemann et al [32] about exposure to images on social media related to the internalization of an ideal thin appearance, causing dissatisfaction with the body and face of the individual. We found a pattern suggesting that the influence depends on the level of self-acceptance of the respondents. With this internalization, if the current body condition is not per the ideal standard and respondents have low self-acceptance, then they will tend to be affected because they have not accepted the current body condition. Conversely, if respondents already have self-acceptance, they tend to be more accepting of all their body conditions and produce a stable level of BS. This is also in line with Rodgers et al [10], who stated that high self-confidence or self-esteem in individuals tends to result in lower levels of dissatisfaction, especially among women.

## Discussion

### Principal Findings

The desire to resemble celebrities seen on TikTok (UAC) is shaped by various activities on the platform, including watching videos, perusing comments and likes, and uploading content (video-based activity). TikTok exposes users to diverse physical appearances that are often perceived as more appealing, encompassing facial features, skin type, color, and body shape. Exposure to such diverse physical attributes prompts unconscious self-evaluation, fostering a longing to emulate those deemed more attractive. This desire is further influenced by the heightened motivation to present oneself attractively on social media (AM). Greater motivation heightens the aspiration to mirror the perceived attractiveness of these individuals [38].

Moreover, this study found the development of TII is influenced by AM, SML, and UAC. The drive to present attractively on TikTok (AM) shapes the perception of an ideal body. Regular exposure to videos emphasizing a “thin body as the ideal” (TII) fosters the internalization of this concept. Conversely, users with a higher level of SML can discern realistic content, thus avoiding the internalization of potentially harmful ideals. Although the TikTok algorithm aids in content curation, personal discernment becomes crucial; failure to discern can lead to increased exposure to harmful content. The aspiration to resemble individuals observed on TikTok (UAC) prompts a comparative self-assessment, often yielding unfavorable outcomes, as users perceive those they watch as possessing more attractive physical attributes. This perpetuates an ideal that equates thinness with attractiveness, further reinforcing TII.

Consequently, the intense desire to emulate others on TikTok (UAC) contributes to a diminished self-assessment, fostering a heightened awareness of one’s perceived inadequacies and resulting in body dissatisfaction. Similarly, strong adherence to thin body standards establishes unrealistic benchmarks, potentially leading to dissatisfaction when one’s appearance fails to meet these standards.

Notably, the shift from the original appearance pressure variable to the AM variable revealed its influence on TII and UAC. Findings from the qualitative analysis indicate that users are motivated by positive comments on content, driving a desire to emulate the praised appearance of content creators, aligning with Trekels and Eggermont [38], who highlighted the reinforcement of appearance-focused media messages through social validation. Furthermore, this study indicates that users’ SML levels impact TII but do not significantly affect UAC. Despite users’ adeptness in discerning social media techniques, they still compare themselves with physically attractive individuals, as seen in their social media feeds. Notably, video-based activity, an original stimulus variable in the TIM, does not significantly impact TII. This contrasts with previous findings [14,26,28], supported by statements from other sources, including research results [50] and statements from social media platforms, such as TikTok [21] and Instagram, signaling a changing trend in the perception of the ideal body, emphasizing self-love and body positivity.

This study establishes the significant impact of organismal variables, namely UAC and TII, on the response variable, BS. Prior research has highlighted the negative impact of comparing oneself with others on social media, leading to decreased BS [9,12,14]. Conversely, individuals exhibiting self-acceptance tend to maintain stable levels of BS [10]. Similarly, previous studies emphasize that internalizing the ideal thin appearance from TikTok content leads to dissatisfaction with one’s physical appearance, while self-acceptance fosters stable BS [10,32].

Practically, these findings offer crucial insights for TikTok, a platform that frequently features content related to physical appearance, aiming to mitigate its potential negative impact on users’ BS. Ong and Sündermann [52] found that a self-guided mHealth app could improve body image concerns and self-compassion in young adult university students. Moreover, a social media–based, fictional 6-episode video series with self-guided web-based activities for improving body image could increase trait BS and mood and decrease internalization of appearance ideals [53]. Therefore, this research encourages the development of a healthier social media environment, fostering users’ mental well-being and comfort.

### Limitations

This study has two limitations. First, respondents mostly live in the greater Jakarta area which is the biggest city in Indonesia. Second, the respondents for this study are predominantly undergraduate students who are either currently pursuing or had completed their undergraduate education. Therefore, the background of the area and current activities of these respondents may impact the results of this study which are only based on the perspectives of students and users in the big cities in Indonesia. Future studies could be carried out to involve respondents from urban areas and other users in smaller cities. However, it is important to acknowledge that these limitations align with a survey conducted by the IDN Research Institute [22]. The survey indicated that over 70% of Gen Z individuals in Indonesia are still in junior high school and senior high school, while the percentage of Gen Z individuals currently pursuing education beyond high school is only 10.36% [22]. Future studies are expected to provide comparisons between the results of this study and studies involving other generations and other social media.

## Appendix

Multimedia Appendix 1 Questionnaire.

Multimedia Appendix 2 Interview questions.

## Abbreviations

AM: appearance motivation
BS: body satisfaction
CA: Cronbach α
CB-SEM: covariance-based structural equation method
Gen Z: Generation Z
SML: social media literacy
SOR: stimulus-organism-response
TII: thin-ideal internalization
TIM: tripartite influence model
UAC: upward appearance comparison
VBA: video-based social media use

## Data Availability

The data sets generated or analyzed during this study are not publicly available due to the lack of authority to share data.
