# Supplementary material for: Influence of TikTok on Body Satisfaction Among Generation Z in Indonesia: Mixed Methods Approach
Source: JMIR Hum Factors. 2024 Sep 6;11:e58371. doi: 10.2196/58371 (PMC11430397; doi:10.2196/58371)
Supplement: Multimedia Appendix 1 [file humanfactors_v11i1e58371_app1.docx]

# Multimedia Appendix 1. Questionnaire.

## A. Video-Based Activity (VBA)

- - - 1. I use TikTok to upload videos related to my physical appearance.
      2. I use TikTok to watch videos related to physical appearance.
      3. I use TikTok to follow accounts that upload videos related to physical appearance (e.g., artists, models, and influencers)
      4. I use the comment feature on TikTok to comment on videos related to physical appearance.
      5. I use the like feature on TikTok to like videos related to physical appearance.
      6. I use the share feature on TikTok to share videos related to physical appearance.
      7. I added videos related to physical appearance to my bookmark list on TikTok.
      8. I use the filter or effect feature on videos related to physical appearance that I upload on TikTok.

## B. Social Media Literacy (SML)

When I watch a TikTok video about physical appearance, I think about the message that video wants to convey (e.g., videos on self-confidence tips, how to take care of yourself, how to look good, and so on)

When I watch a TikTok video that shows someone's physical appearance that looks perfect, I think that it is unrealistic (for example, a video using filters or effects)

When I watch a TikTok video that shows someone's physical appearance, I wonder if the purpose of the video will be good for me.

When I watch a TikTok video about how to look more attractive, I assess whether the delivery method of the video is right or wrong.

## C. Appearance Motivation (AM)

- - - 1. I feel motivated to look more fit after watching videos related to physical appearance on TikTok.
      2. I feel motivated to have an ideal body after watching videos related to physical appearance on TikTok.
      3. I feel motivated to have a better physical appearance (according to my preference) after watching videos related to physical appearance on TikTok.
      4. I feel motivated to lose weight after watching videos related to physical appearance on TikTok.

## D. Thin-Ideal Internalization (TII)

I imagine to look thin after watching videos related to physical appearance on TikTok.

I have a desire to look thin after watching videos related to physical appearance on TikTok.

I try to look thin after watching videos related to physical appearance on TikTok.

I often thought the thin body looked more attractive after watching videos related to physical appearance on TikTok.

## F. Upward Appearance Comparison (UAC)

1. I compare my physical appearance with the people I saw on videos on TikTok when their physical appearance is more attractive.
2. I wonder how my physical appearance becomes when it compares to the physical appearance of influencers, artists, and models on TikTok.
3. I think my physical appearance is as good as the physical appearance of the influencers, artists, and models on TikTok.
4. When I read praise comments related to other's physical appearance on TikTok, I compare my physical appearance with the person who received the compliment.
5. When I read comments praising other people's physical appearance on TikTok, I wonder if I could get that when I uploaded the video.

## G. Body Satisfaction (BS)

- - - 1. I feel satisfied with my own body after watching many videos related to physical appearance on TikTok.
      2. I am encouraged to value my own body by adopting a healthy lifestyle after watching many videos related to physical appearance on TikTok.
      3. I feel more confident after watching many videos related to physical appearance on TikTok.
      4. I feel good about my own body after watching many videos related to physical appearance on TikTok.

1. I feel happy when videos related to physical appearance on TikTok convince me that my body is incredible.
2. I consider my body perfect after watching many videos related to physical appearance on TikTok.
3. I feel more confident about my body after reading positive comments from others on videos I uploaded about my physical appearance on TikTok.
