# Supplementary material for: Influence of TikTok on Body Satisfaction Among Generation Z in Indonesia: Mixed Methods Approach
Source: JMIR Hum Factors. 2024 Sep 6;11:e58371. doi: 10.2196/58371 (PMC11430397; doi:10.2196/58371)
Supplement: Multimedia Appendix 2 [file humanfactors_v11i1e58371_app2.docx]

# Multimedia Appendix 2. Interview questions.

| **Hypothesis** | **Questions** |
| --- | --- |
| H1 | What do you think about the physical appearance videos that you often see on TikTok?  In your opinion, how does content related to the physical appearance of thin people on TikTok influence the way you view the meaning of an ideal body?  Do activities such as viewing, uploading, or interacting with videos showing your physical appearance on TikTok make you want to change your physical appearance to be thinner? If not, why? |
| H2 | When you watch a video related to physical appearance, do you try to judge the person in the video?  Have you ever judged that the physical appearance of the person you see on TikTok is more attractive than your appearance? Explain why.  Does this make you compare your physical appearance to the people you see in videos?  In your opinion, how do the activities related to physical appearance that you do on TikTok affect your self-evaluation? |
| H3 | From the various physical appearance videos, you see on TikTok, have you been influenced to improve your physical appearance to become thinner? Explain the reasons why this might happen. |
|  | Have you ever tried editing or using filters on videos of your physical appearance to make your body look thinner? Explain why you edit or use filters on your videos. |
|  | How would you respond if the physical appearance video you saw was an edited video to make the person look thinner? |
| H4 | From the various physical appearance videos that you see on TikTok, have you ever compared your physical appearance with someone who you think has a better physical appearance than you? If not, why? |
|  | Have you ever tried editing or using filters on videos of your physical appearance to make them look more attractive like the people you see on TikTok? If not, why? |
|  | How do you respond to TikTok videos that show less realistic physical appearance regarding your self-assessment? For example, videos that use filters to make the person look attractive, like a model or celebrity. |
| H5 | From the various physical appearance videos, you see on TikTok, have you ever been motivated to look thinner? Explain why. |
|  | If you have ever been motivated to look thinner, what things can support you in achieving your goal? |
| H6 | From the various physical appearance videos, you see on TikTok, have you ever been motivated to have a physical appearance like a model or health influencer? |
|  | Have you ever been motivated to look better on TikTok to be considered equal to people whose physical appearance you consider more attractive on TikTok? Explain why. |
| H7 | If you have ever compared your physical appearance with someone who has a thinner physical appearance, explain how much influence this has on your perception of an ideal body? |
|  | If you have ever compared your physical appearance with someone who has a thinner physical appearance, how do you feel after being compared? |
| H8 | If you have ever compared your physical appearance with someone who has a more attractive physical appearance, how does this affect your self-satisfaction with your own physical appearance? |
|  | In your opinion, if someone compares their physical appearance with other people who have a more attractive physical appearance, is that person not satisfied with their current body shape? Explain why. |
| H9 | If you feel that the ideal body is a thin body, how does this thought affect your self-satisfaction with your physical appearance? |
|  | In your opinion, if someone has the desire to look thinner, is that person not satisfied with their current body shape? Explain why. |
